# A convolutional neural network based online teaching method using edge-cloud computing platform

**DOI:** 10.1186/s13677-023-00426-6

**Published:** 2023-03-28

**Authors:** Liu Zhong

**Affiliations:** Shandong University of Arts, Jinan, China

**Keywords:** Edge calculation, Dance action correction, Grid coding, Key frame extraction

## Abstract

Teaching has become a complex essential tool for students’ abilities, due to their different levels of learning and understanding. In the traditional offline teaching methods, dance teachers lack a target for students ‘classroom teaching. Furthermore, teachers have limited time, so they cannot take full care of each student’s learning needs according to their understanding and learning ability, which leads to the polarization of the learning effect. Because of this, this paper proposes an online teaching method based on Artificial Intelligence and edge calculation. In the first phase, standard teaching and student-recorded dance learning videos are conducted through the key frames extraction through a deep convolutional neural network. In the second phase, the extracted key frame images were then extracted for human key points using grid coding, and the fully convolutional neural network was used to predict the human posture. The guidance vector is used to correct the dance movements to achieve the purpose of online learning. The CNN model is distributed into two parts so that the training occurs at the cloud and prediction happens at the edge server. Moreover, the questionnaire was used to obtain the students’ learning status, understand their difficulties in dance learning, and record the corresponding dance teaching videos to make up for their weak links. Finally, the edge-cloud computing platform is used to help the training model learn quickly form vast amount of collected data. Our experiments show that the cloud-edge platform helps to support new teaching forms, enhance the platform’s overall application performance and intelligence level, and improve the online learning experience. The application of this paper can help dance students to achieve efficient learning.

## Introduction

In recent years, due to COVID-19, teaching methods have had to be transferred from offline to online [1–3]. However, college students come from different countries and regions, with the characteristics of the large number, wide distribution area, and complex personnel composition. Moreover, for students majoring in dance, online teaching can only learn the dance movements by watching the dance teaching videos recorded by the teacher in advance, which cannot accept the correction of the teacher’s movements face to face, and also cannot intuitively feel the essentials of each dance movement. Therefore, this paper presents the online dance teaching method based on AI and edge calculation. AI is used to make up for the shortcomings of traditional teaching methods. The edge intelligent services such as computing and storage provided on the network edge side near objects or data sources are used to improve the application performance and intelligence level of a network teaching and the teaching experience.

The online dance teaching platform of this paper involves many high-definition teaching videos. At the same time, the identification of dance movements, the extraction of key frames, and the online correction of dance movements all need real-time and accurate data transmission. Therefore, this paper mainly uses the following advantages of edge computing: (1) Complete the data processing near the data source to reduce the transmission delay and improve the data processing efficiency; (2) Compared with cloud centers, edge computing, and storage costs are lower; and (3) Low dependence on the cloud, which can reduce the data transmission error rate and single point of failure rate.

This paper proposes an online dance learning method based on "video key frame extraction + human key point position extraction + action correction," which can identify and correct dance movements during estimation. The proposed model is implemented over the edge-cloud platform so that the training happens at the cloud while the prediction and correction happens at the edge. The edge computing mode can effectively reduce the transmission delay of network dance teaching content, provide students with richer and faster teaching content, and significantly improve the online learning experience. This paper provides a reliable basis for exploring the information and efficient mode of dance teaching. The major contributions of this paper are as given below:

- we propose an online dance learning method based on "video key frame extraction + human key point position extraction + action correction," which can identify and correct dance movements during estimation;
- the proposed model is implemented over the edge-cloud platform so that the training happens at the cloud while the prediction and correction happens at the edge; and
- we test the model using datasets and questionnaire survey and report our major observations and outcomes.

The remaining part of the paper is organized in the following manner. A brief summary of the related work is deliberated in Sect. "Related Work". In Sect. "An introduction to the convolutional neural network", we offer a brief review of the convolutional neural network (CNN). In Sect. "Video key frame extraction based on a deep convolutional neural network algorithm", we discuss video key frame extraction based on a deep convolutional neural network algorithm. The algorithm results are discussed in this section. In Sect. "Dance action correction process", we describe the grid coding, through which the critical positions of the human body are determined and extracted. We discuss an algorithm that detect the key positions in the image using the convolutional neural network. In Sect. "Dance teaching method based on knowledge concealment", we discuss the validation of the proposed mode. In Sect. "The Design of an Online Teaching System Based on Edge Computing", the CNN model is distributed over two servers (cloud and edge) where some layers are running on the cloud for feature extraction and training while other layers run over the edge cloud. The results are explained in terms of training and prediction durations. Finally, Sect. "Conclusions" concludes this paper.

## Related work

In this section, we briefly discuss some of the related work in terms of techniques which are based on the AI and machine learning.

### The dance teaching method based on AI

The method involves two key steps: video key frame extraction and dance action correction. The former is mainly designed to extract the key frames in the dance movements, while the latter is mainly designed to extract the human key points in the image and then correct the movements by the guidance vector. For the video, its main component unit is the frame, and a series of continuous image frames in time form the video. Therefore, these key frames must be extracted to reflect the video’s main characteristics. At present, the common key frame extraction methods mainly include the key frame extraction algorithm based on SIFT features [4, 5], the clustering-based key frame extraction algorithm [6, 7], and a key frame extraction algorithm based on motion analysis [8]. However, the traditional algorithm has a low accuracy rate for complex dance teaching video extraction. This paper proposes a deep convolutional neural network algorithm to extract the key frames of videos and use the pre-trained CNNs network model.

At present, the common key frame extraction methods mainly include the key frame extraction algorithm [4, 5] based on SIFT features, the clustering-based key frame extraction algorithm [6, 7], and [8], a key frame extraction algorithm based on motion analysis, however, in the key frame extraction algorithm based on SIFT features in the video key frame extraction. The smooth edge targets cannot accurately extract the feature points and have poor real-time performance, thus affecting the integrity and effectiveness of the key frame extraction [9, 10]. Clustering-based key frame extraction algorithm generally needs to set the center and number of clusters in advance in the clustering process. Still, the center and number of clusters should not be determined in practical applications. Moreover, in the clustering process, the original image frames’ time order and dynamic information in the original lens need to be better retained. The video frames not in one lens or scene will be clustered together. The video index needs to be better established in video retrieval to reduce the accuracy of video retrieval [11]. The key frame extraction algorithm based on motion analysis requires much effort to calculate the amount of exercise. The local minimum determination is inaccurate, reducing the algorithm’s efficiency. Sometimes, it also causes the miscalculation of key frames. The resulting key frame cannot better express the summary information of the video [12]. This paper proposes a deep convolutional neural network algorithm to extract the key frames of videos and use the pre-trained CNNs network model to learn the new teaching characteristics.

In addition to key frame extraction, this paper extracts the human pose using grid coding and then corrects the human posture using the guidance vector. Human pose estimation and correction are widely used in computer vision and can mainly conduct human–computer interaction, motion analysis, and action recognition [13]. Although artificial intelligence coaches can guide the human body’s movements, dance major students can correct their movements through artificial intelligence coaches. But professional equipment is often expensive and difficult to use online. Therefore, the grid coding and guidance vector combination can correct the students’ dance movements based on finding the key positions in the human body. This reduces the equipment’s input cost and improves the correction’s accuracy.

### Applications of edge computing in resource integration of teaching platforms

Edge computing is a system architecture under the cloud computing framework, with computing, caching, and local application functions by deploying edge node devices at the network edge side close to the data source [14–16]. Edge nodes collaborate with the cloud center to support real-time business, agile connectivity, and application performance optimization and change the way data is centrally stored and processed in the traditional network architecture.

They were using the edge node equipment, such as the gateway, base station, server, and intelligent terminal close to the data source end, to complete part of the data storage and processing tasks originally undertaken by the data center. Usually, data is only used locally, and business processing requires low latency and fast response. Security-sensitive data are put on edge devices to improve the service response speed, ensure data security, and provide a better user experience [17–19]. Some researchers have used the splitting mechanism for the machine learning models such as CNN so that some of the layers can run on the cloud and some on the edge servers. The layers that need more computational power can be executed on the cloud such as feature extraction and training while the layers that do not need much compute resources such as prediction and data collection might be run on the edge [20–25]. However, this kind of distribution might be difficult and will vary from application to application.

## An introduction to the convolutional neural network

**Fig. 1:**
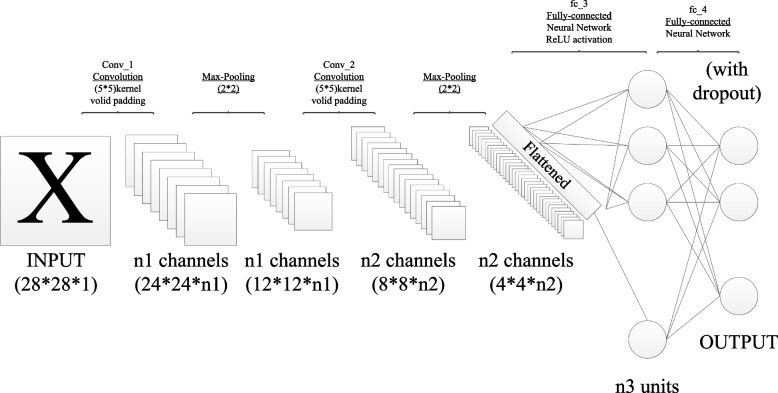
Convolutional neural network structure

Figure 1 is a typical convolutional neural network (CNN) structure, consisting of an input layer, a convolutional layer, a subsampling layer (a pooling layer), a full connection layer, and an output layer.

The input of the CNN is usually the original image $X$, If ${H}_{i}$ is a characteristics graph of the convolutional neural network layer $i\left({H}_{0}=X\right)$, the generation process of ${H}_{i}$ is as follows:

1 $${H}_{i}=f\left({H}_{i-1}\otimes {W}_{i}+{b}_{i}\right)$$

where: ${W}_{i}$ is the weight vector of the first-level convolution kernel $i$, the symbol $\otimes$ represents the convolution process of convolution kernel with images of $i-1$ or feature maps. The output of the convolution is added to the offset vector ${b}_{i}$ at the level of the $i$ layer. Finally, the feature plot ${H}_{i}$ of layer $i$ is obtained by the nonlinear excitation function $f\left(x\right)$. Subsampling layers are usually behind the convolution layer, and the subsampling rules are as follows:

2 $${L}_{i}=subsampling\left({H}_{i-1}\right)$$

The CNN classifies the extracted features by alternating transmission of multiple convolutional layers and lower sampling layers to obtain a probability distribution based on the input $Y$.

3 $$Y\left(i\right)=P\left(L={l}_{i}\left|{H}_{0};\left(W,b\right)\right.\right)$$

The CNN is a common gradient descent method during training. Residuals are propagated by gradient descent and the trainable parameters of each layer of CNN are updated ($W$ and $b$). The learning rate parameters are used to control the strength of the normal propagation of the residuals:

4 $${W}_{i}={W}_{i}-\eta \frac{\partial E\left(W,b\right)}{\partial {W}_{i}}$$

5 $${b}_{i}={b}_{i}-\eta \frac{\partial E\left(W,b\right)}{\partial {b}_{i}}$$

## Video key frame extraction based on a deep convolutional neural network algorithm

In video key frame extraction, the video key frame can be extracted in units and the original video as the starting point. This section’s key frame extraction method is based on the original video. Because the convolutional neural network has powerful learning ability, it can mine the intrinsic implicit relationship of the training video frame image. Use its internal feature collector to extract the underlying high-level features of the video frame, normalizing the frame image features across scales. Finally, obtain the significant feature vector of the video frame image. Using the frame similarity measurement algorithm to get the frame similarity value, the judge in an adaptive similarity measure threshold and, finally, the key frame of the original video sequence.

**Fig. 2:**
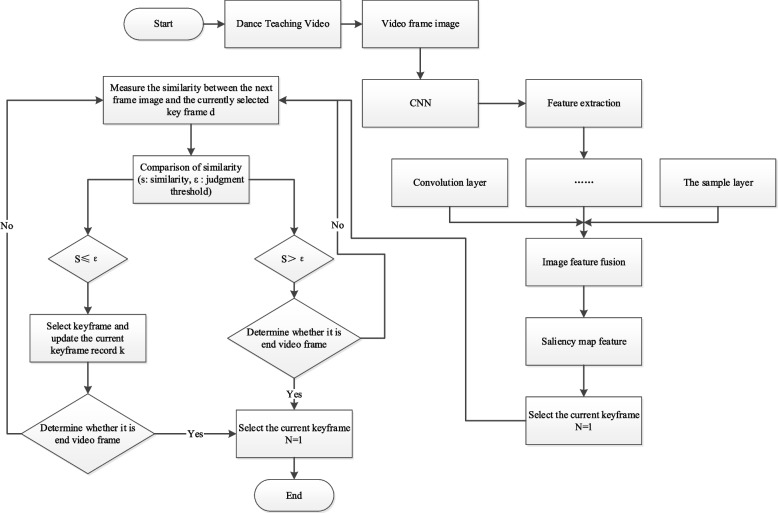
Flow chart of the key frame extraction algorithm based on convolutional neural video

Figure 2 shows a flow chart of the algorithm.

### Video frame image feature extraction

This section uses the deep learning network model where the feature extraction happens at the remote cloud due to the need for high computational power. The deep convolutional neural network (CNN) model has good advantages in obtaining the intrinsic features of frame images (underlying features to top semantic features) of frame images and shows good performance in other applications (such as object detection, image classification, etc.). The feature extraction process of the deep convolutional neural network is as follows:

1. Convert the original video into a frame set according to the original spatial and temporal sequence. The video frame image size is 128*128.
2. Set the convolutional neural network, and adopt the C-S network model structure as the basic architecture because the CNNs can extract the deep features of the images, which can get better results than using other networks to extract the image features. The convolutional network contains two large layers (C layer), two subsampling layers (S layer), and two fully connected layer outputs.
3. In the convolutional layer C1 layer, the video frame is mainly a convolutional operation, which uses three 3*3 convolution cores. The Convolutional kernel is generated through random data, combining the basic principles of backpropagation and forward propagation, and finally, reverse adjusts its parameter information according to the obtained results. The size of the internal neuron receptive field is the size of the adjustment. If the receptive neuron field size is too small in the adjustment process, extracting the video frame image features will be insufficient. At the same time, the extracted features will be too complex. This layer is to extract some primary underlying features of the video frames. Three 124*124 size feature maps are output after the layer.
4. In the S1 layer of the subsampling layer, the feature map output by the C1 layer is mainly processed, and the sampling window size is set as 2*2. The mean-pooling method is used better to retain the original feature information of the image. Through the down sampling processing of this layer, the feature map output by the C1 layer is processed as a 62*62 size feature map. This location improves the sampling of image features while reducing the dimension.
5. The convolution layer C2 of the second layer has a 5 features diagram. The convolution check image of 3*3 is still used for convolution operation and outputs the feature image of size 58*58. In the second subsampling layer, S2 also uses the original window size and outputs five feature maps of 29*29. However, these two layers can be used to extract more differentiated image features.
6. Video frame features extracted from different convolutional kernels in deep convolutional neural networks. When integrating these features, you can not only integrate the features of multiple video frames but also maintain the information of the original video frame. In addition, the video frames obtained through fusion have almost rich features and more clear colors. The linear fusion method that was used is, as shown in Eq. (6):
  6 $$F=\alpha \times {\mathrm{S}}_{i}^{1}+\beta \times {\mathrm{S}}_{i}^{2}+\delta \times {\mathrm{S}}_{i}^{3}+\cdots \cdots +\eta \times {\mathrm{S}}_{i}^{n}$$
  Where F is eigenvectors after the fusion, the sum of coefficient $\alpha$ and $\beta$ is 1, ${S}_{i}$ is the output feature image corresponding to each layer.

### Interame similarity measures

A similarity measurement algorithm describes the correlation or similarity between two things. It is widely used in information retrieval, image recognition, document classification, and other fields. Inter-frame similarity measurement algorithms can confirm the acquaintance of video frames and provide a metric for key frame extraction in content-based video retrieval. This section selects the similarity measure of the front and rear frames in the video, and a modified Euclidean distance algorithm is used to calculate the similarity between the frames, as shown in Eq. (7):

7 $${S}_{t}\left({F}_{i},{F}_{j}\right)=1-{\left\{\sum_{t-1}^{m}\left[{\left({\mu }_{t}^{{F}_{i}}-{\mu }_{t}^{{F}_{j}}\right)}^{2}+{\left({\lambda }_{t}^{{F}_{i}}-{\lambda }_{t}^{{F}_{j}}\right)}^{2}\right]\right\}}^{1/2}$$

where ${F}_{i}$ and ${F}_{j}$ characterizes all the immediately adjacent video frames in the original video. Moreover, ${\mu }_{t}^{{F}_{i}}$ and ${\lambda }_{t}^{{F}_{j}}$ are the normalized feature vectors obtained after feature extraction in video frame images, and $m$ is the dimensions of the extracted features. Note that the smaller value for $S$ indicates a lower similarity measure of two frames, and the higher the image similarity of two frames.

### Key frame extraction

When extracting a video key frame based on a deep convolutional neural network, the original video is taken as the whole unit, rather than splitting the video into lenses and extracting the key frame. The basic steps of this algorithm are:

1. Using the powerful advantages of convolutional neural networks for extracting video frame image features under non-artificial intervention. The convolutional layer, pooling layer, down sampling layer, and full connection layer can extract the underlying and deep features of the frame image, respectively. Then these features are integrated to highlight the salient feature map of the frame image.
2. From the first frame of the video, preset as a key frame, and then the similarity S is calculated according to the inter-frame similarity measurement algorithm formula (3). Use the next frame and the current selected key frame for the similarity comparison. If S is the similarity contrast is less than a certain threshold, put the frame into the key frame sequence, and then record the position encoding of the frame in the original video. On the contrary, if the S in the similarity comparison is greater than or equal to a certain threshold, continue to make the similarity comparison between the next frame of the current key and the video.
3. In making the similarity measurement, to make the algorithm better scalable, the limit value should be adjusted appropriately according to the different types and complexity of the video. With the threshold setting effect, try to extract the video key frame while reducing redundancy. An adaptive threshold determination method is used to set the threshold value.
  8 $$\varepsilon =\frac{1}{n}\sum_{i-1}^{n}S\left({F}_{i+1},{F}_{i}\right)-\tau$$
  Where $\upvarepsilon$ is a threshold quantity, and $\tau$ is the adaptive adjustment number of the limit value.

(4) At the end of the video frame scanned by the method of steps (2) and (3), to end the extraction of the video key frame. Finally, a set of key frame with temporal characteristics (sequence set greater than or equal to 1).

### Experimental results of the algorithm

To highlight the advantages of this algorithm, dance teaching videos of different lengths were selected for analysis and explanation during the experiment. And recall ratio R and accuracy ratio P (as shown in formulas (9) and (10)) are used to measure the experimental results of the extraction of the video key frame by the algorithm. Then, compared with the existing video key frame extraction algorithm, further explain the feasibility and effectiveness of the proposed algorithm:

9 $$\left(\mathrm{Re}call\right)R=\frac{{N}_{C}}{{N}_{C}+{N}_{M}}$$

10 $$\left(\mathrm{Pr}ecision\right)P=\frac{{N}_{C}}{{N}_{C}+{N}_{F}}$$

where ${N}_{C}$ is the number of extracted video key frame, ${N}_{M}$ is the number of missed video key frame, and ${N}_{F}$ is the number of missed video and video key frame.

**Fig. 3:**
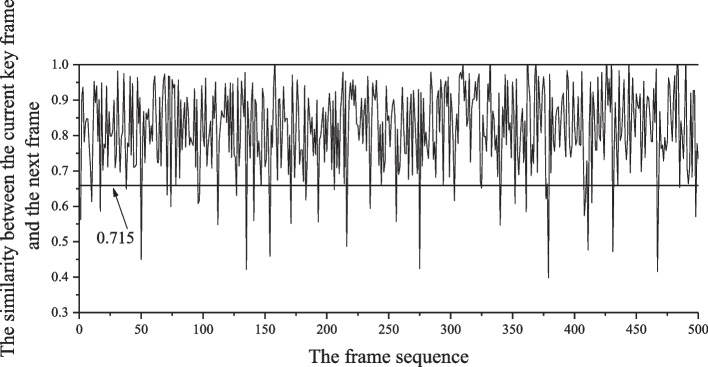
The Interame similarity measurement plot

First, the short dance teaching video is selected, and the present algorithm is used to analyze the key frame extraction experiment. The video is 321 s long and contains 2027 frame images (extracted frame rate of 10 frames). The video is called "Basic Skills Teaching of Dance," and it is mainly about the basic skills teaching of dance. The video content is relatively complex, and the camera changes quickly, which is very persuasive in the video key frame extraction experiment. Using this algorithm in the experiment process of extracting key frames, an adaptive method is adopted to set the threshold value of $\varepsilon$. The adaptive adjustment number of the first setting of the threshold value of $\tau$ is set to 0.137. Then, the adaptive threshold value $\varepsilon$ is 0.715 according to formula (3). Figure 3 shows a similarity measure graph between the first 500 frames to confirming whether the frame is added to the key frame sequence by measuring the relationship between the current key frame and the next video frame. If yes, then we update the key frame sequence set, otherwise we discard this, and continue comparing the next video frame until the last frame of the video.

**Fig. 4:**
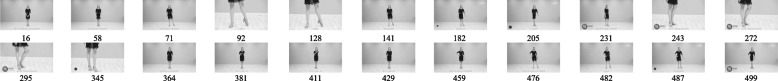
Key frames of the first 500 video frames

**Fig. 5:**
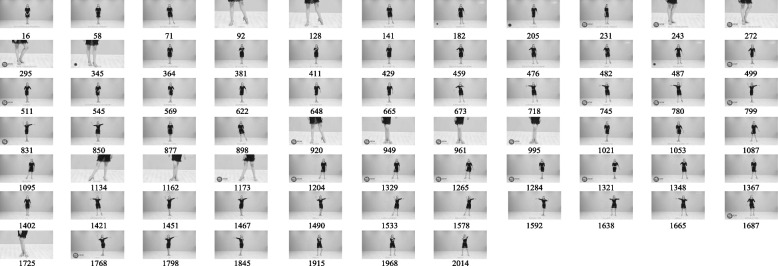
Overall key frame

This method shows the obtained experimental results in Fig. 4, and the first 500 video frames are first selected for key frame extraction. Figure 5 shows the key frame sequence of the dance teaching video obtained using the same method to verify the original video based on selecting the key frames in the first 500 frames.

The following experiment uses the algorithm and the existing popular video key frame extraction algorithm to compare and analyze video clips of different lengths. Table 1 gives the detection results of the video key frame extraction, where A represents the present method, B represents the SIFT method, and C represents the clustering method.

**Table 1:** Comparative analysis of key frame extraction results of different algorithms

	274 s			753 s			1437 s		
	A	B	C	A	B	C	A	B	C
Total frame number	6325			15,479			31,884		
Key frame number	98	98	98	440	440	440	884	884	884
Extract the quantity	98	95	99	438	434	446	883	880	887
N_C_	91	82	81	424	388	374	857	783	791
N_F_	7	13	18	13	46	72	26	97	97
N_M_	7	15	16	15	52	66	27	101	93
R/%	92.86	86.32	81.82	96.80	89.40	83.86	97.06	88.98	89.18
P/%	92.86	83.67	82.65	96.36	88.18	85.00	96.95	88.57	89.48

According to Table 1: The proposed method has better results in a video of different lengths than the traditional SIFT-based and clustering-based methods. For example, the recall rate of this algorithm in 274 s, 753 s, and 1437 s dance videos is 92.86%, 96.80%, and 97.06%, respectively, which is higher than the other two methods, and the recall algorithm in this paper is also higher than the traditional classical methods. Therefore, through the experimental comparative analysis, the proposed video key frame extraction algorithm based on the deep convolutional neural network has high accuracy, and the extracted key frames can better express the summary information of the video.

## Dance action correction process

Based on the grid coding, the critical positions of the human body in the key frame image are determined and extracted. In the next phase, the algorithm detects the key positions in the image using the convolutional neural network. Finally, the dance movements are corrected by the guidance vector. The specific implementation process is as follows.

### Grid coding process

This should be noted that ${T}_{i}=\left\{{t}_{1},{t}_{2},\dots {t}_{n}\right\}$ is the location of class N human points of the human body in the image. Take class n human key-point position ${t}_{i}=\left\{{x}_{n},{y}_{n}\right\}$ as an example, the grid encoding process presented in this paper is as follow:

1. Divide the image into s*s to determine which grid the human key points appear.
2. Write in 3 messages in per grid, the probability of human key points ${p}_{n}$, horizontal offset ${\overline{x} }_{n}$ relative to the upper left corner of the grid, and vertical offset ${\overline{y} }_{n}$ relative to the upper left corner of the grid.
3. If a grid that does not contain human key points, then its probability $\left({p}_{n},{\overline{x} }_{n},{\overline{y} }_{n}\right)$ is 0. Furthermore, if a grid containing the key points of the human body, then its probability ${p}_{n}$ is 1. The ${\overline{x} }_{n}$ and ${\overline{y} }_{n},$ is normalized to [0,1]. The calculation process is shown as follows in Eqs. 11 and 12:

11 $${\overline{x} }_{n}=\frac{{x}_{n}-{x}_{g}}{b}$$

12 $${\overline{y} }_{n}=\frac{{y}_{n}-{y}_{g}}{b}$$

**Fig. 6:**
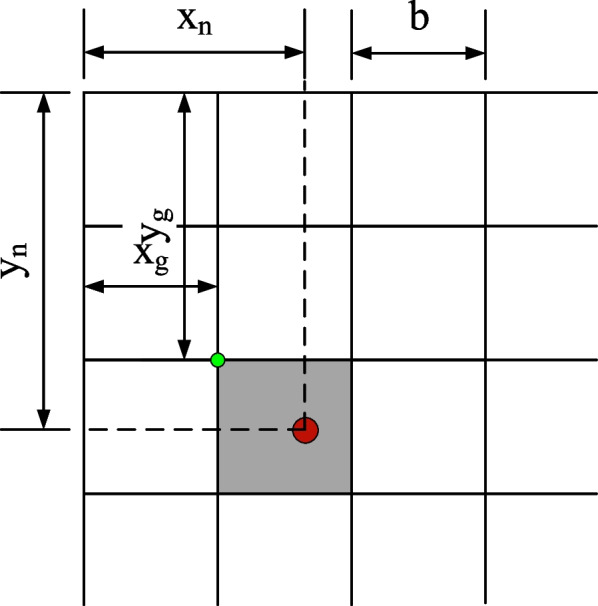
Grid-encoding positional relationships

Where, the variable ${x}_{g}$ is the horizontal position in the upper left corner of the grid, variable ${y}_{g}$ is the vertical position in the upper left corner of the grid, and variable $b$ is the grid side long. The specific meaning can be understood from Fig. 6. The red point indicates the key point position of the human body and falls in a network, and the green point is the upper left corner of the grid (Fig. 6).

**Fig. 7:**
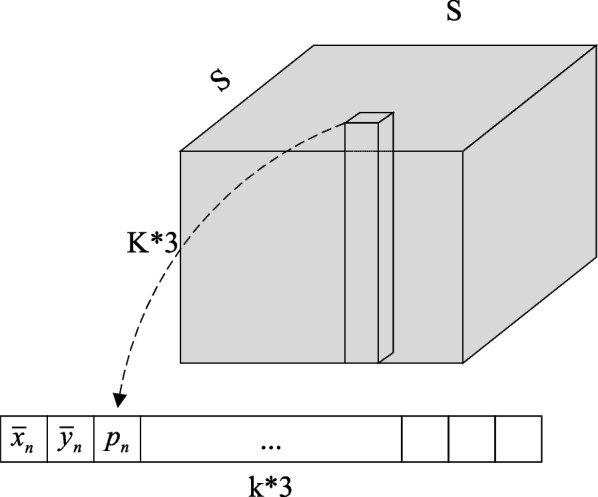
Grid-encoding positional relationships

Suppose that a body contains N key points, in order to distinguish the key categories, each type of human key points needs to be coded separately $\left({p}_{n},{\overline{x} }_{n},{\overline{y} }_{n}\right)$. Therefore, the data obtained by encoding the key point position of the human body is a tensor of the s*s*(N*3) shape. As shown in Fig. 7, it is spatially divided into s*s, the number of channels is N*3, and each spatial position point contains N class human key point information. For the full convolutional neural network, it only needs to set its final output shape to match the tensor shape obtained by encoding the key point position of the human body, and then after the label is supervised training, the full convolutional neural network can obtain the ability to predict this data. When calculating the position of human key points, determine which grid of human key points to appear according to the probability ${p}_{n}$ of each type of human key points, and then roughly locate the key points of the human body. Finally, the human key point position is precisely located according to the output of the grid $\left({\overline{x} }_{n},{\overline{y} }_{n}\right)$.

### Convolutional neural network design for single-person pose estimation

**Fig. 8:**
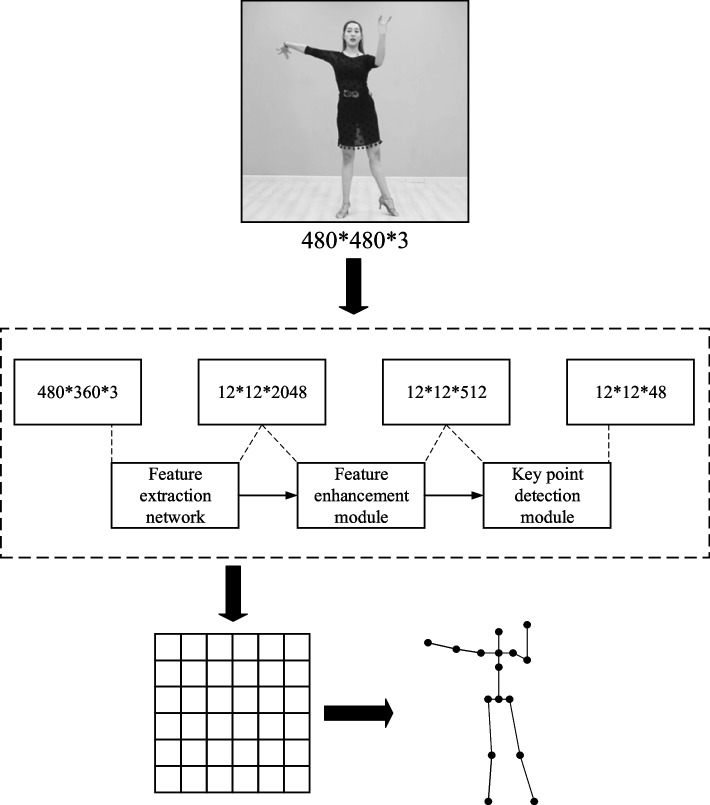
Flow chart of the human pose estimation algorithm

After introducing human key position, a single-person pose estimation algorithm based on grid coding, in addition to the grid coding in the previous section, this section mainly explains and analyzes the convolutional neural network design of the one-person pose estimation algorithm and the relevant techniques of the algorithm prediction process. The flow framework of the overall algorithm for single-person pose estimation based on grid coding is shown in Fig. 8.

The single-person pose estimation convolutional neural network adopted in the present chapter algorithm is fully convolutional. The last layer is a tensor with an output size of s*s*(N*3). The structure can be divided into the feature extraction network, feature enhancement module, and key point detection module. The three-part structure with a sequential connection to form the fully convolutional network is shown in the dashed box in Fig. 8, and each module marks the channel size of the input and output.

The feature extraction network can be designed based on the classification network. Take the residual network ResNet50 as an example to remove the last pooling layer and the full connection layer as the feature extraction network. Images were computed through a feature extraction network to generate 2048-channel abstract features with spatial dimensions of 1/32 times that of the original image size. The ratio of the output feature map size of the convolutional neural network is called the down sampling multiple. The size of the algorithm partition grid s*s in this chapter is determined by the size of the output of the last layer after the down sampling. Take the 384*384 size input image as an example. When the current sampling multiple is 1/32, the output size of the last layer is the divided grid size is 12*12. In addition, the number of output channels of the key point detection module is related to the number of human key point categories. Assuming that the human body has 16 key points, output channels are 16*3.

**Fig. 9:**
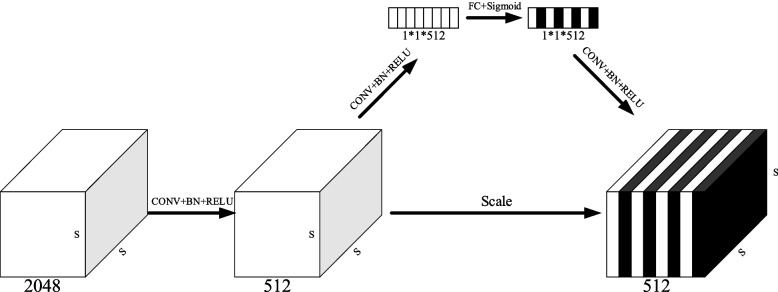
Feature enhancement module structure diagram

The 2048 channel feature is reduced to 512 channels to enhance the channel features while reducing the computational amount. The feature enhancement module is shown in Fig. 9, whose lower branch is convolved by 3*3, batch normalization, and RELU correction of the linear cell activation function composition, reducing the number of channels to 512. The spatial information of the aggregated features is a 512-dimensional vector through the global pooling layers. After two nonlinear maps of the fully connected layers, they are characterized as different channel feature importance with the Sigmoid activation function. Finally, the scale has multiplied the 512 channel features by bit enhancement.

**Fig. 10:**
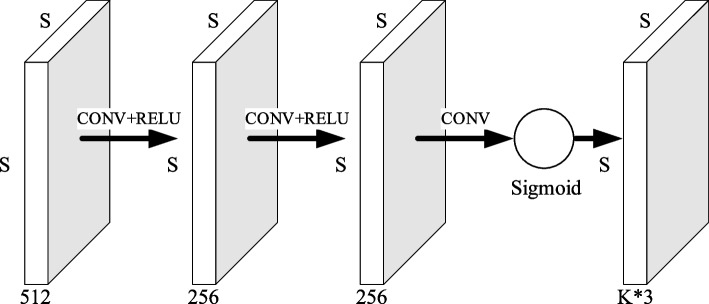
Key point detection module structure diagram

The key point detection module consists of 3 layers of convolution plus an activation function, as shown in Fig. 10. The first two layers of the activation function are the RELU activation function, and the last layer is the Sigmoid activation function, which is designed to limit the output range between (0,1). This is because it needs to learn that the values of the fitted target $\left({p}_{n},{\overline{x} }_{n},{\overline{y} }_{n}\right)$ are all in that range. The output tensor size of the key point detection module is consistent with the tensor size generated by encoding the key point position in the human body, it is s*s*(N*3).

Using the algorithm proposed in this paper, the entire process identifies human key points using all the images extracted from key frames as the test set. As seen from Fig. 3, the extracted key frame images involve different parts of the human body so that they can verify the effect of the key point extraction more effectively. The prediction effects of different human key points are shown in Table 2.

**Table 2:** The prediction results of different human key points (unit: %)

Part	Average	Head	Shoulder	Elbow	Hand	Buttocks	Knee	Foot
Accuracy rate	90.38	98.13	98.20	96.48	91.24	86.69	90.57	86.57

### Dance movement correction based on guidance vectors

Starting from a certain frame of the extracted key frame, the teacher’s action is globally aligned with the key point information extracted from the student’s action image. Here, the mathematical notation (as given by Eq. 13) represents the posture sequence of the teacher’s action and the student’s action.

13 $${\overline{x} }_{n}=\frac{{x}_{n}-{x}_{g}}{b}$$

The next step is to correct the students’ wrong actions by passing the guidance vector (as given by Eq. 14) over the guidance vector.

14 $${\overline{x} }_{n}=\frac{{x}_{n}-{x}_{g}}{b}$$

**Fig. 11:**
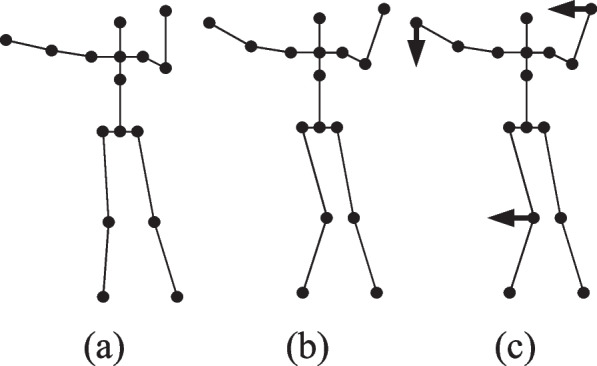
Dance action correction figure

The example diagram is shown in Fig. 11, wherein Fig. 11(a) is a standard action of the teacher and Fig. 11(b) is an action of the student. Similarly, Fig. 11(c) is the result of instructing the students’ incorrect movement of the joints. Through the correction of the action, the students can clearly see their action which is in fact not in place, and then let the students correct themselves.

## Dance teaching method based on knowledge concealment

### Questionnaire design

This paper summarizes 10 indicators from the two levels of dance foundation and advancement to evaluate the effect of dance learning on the students, providing data support for formulating the dance teaching methods and mechanisms based on knowledge hiding. The questionnaire content is shown in Table 3.

**Table 3:** The Dance Learning Effect Questionnaire

Number	Question	Option				
Dance foundation	Keep up with the teacher’s classroom teaching rhythm	5	4	3	2	1
	Master the basic techniques of dance	5	4	3	2	1
	Clarify the movement principle of the dance	5	4	3	2	1
	Rescore the teacher’s demonstration	5	4	3	2	1
	Control the strength of the action	5	4	3	2	1
Dance advanced	Dance movements are coordinated with the musical rhythm	5	4	3	2	1
	You can find out where your movements are not standard	5	4	3	2	1
	Be able to complete a dance independently	5	4	3	2	1
	Dance in the management of emotions and facial expressions	5	4	3	2	1
	Have the consciousness of dance movement innovation	5	4	3	2	1

### Examination of the questionnaire data

#### Reliability test

The SPSS software analyzes the relevant data. Before analyzing the questionnaire data, the reliability of the questionnaire is the reliability test. The reliability analysis method used in this paper is the α reliability coefficient method. From Table 4, the value of Cronbach’s alpha is 0.768, greater than 0.7, indicating that the questionnaire data has high reliability and the questionnaire design is reasonable and can be used as a data source.

**Table 4:** Questionnaire reliability statistics table

Alpha	Clone Bach Alpha based on the normalization term	number of terms
0.866	0.768	27

#### Validity test

The questionnaire’s validity test applied the KMO test to check the partial correlation between the variables. As can be seen in Table 5, the KMO value of this questionnaire data test was 0.757, and the value was more significant than 0.7. The approximate chi-square of the Batley spherical test is 3847.628, the degrees of freedom are 279, and the significance is 0.000, less than 0.05, so the questionnaire scale can be determined to have sufficient validity.

**Table 5:** Questionnaire validity test form

KMO and Bartlett tests		
Number of KMO sampling suitability	.757	
Batley Sphicity Test	Approximate chi square	3847.628
	Free degree	279
	Conspicuousness	.000

### Questionnaire results

The respondents of this questionnaire were dance students, including 429 students from freshman to senior year. A total of 429 questionnaires were sent out, 408 were collected, and 396 valid questionnaires with an effective rate of 92.3%. For the 10 questions in the questionnaire, options 1–5 correspond to 1–5 points, respectively, and a higher total score indicates a better learning effect. The scores of questions 1–10 are shown in Table 6 (partial results).

**Table 6:** Questionnaire Results (partial results)

Number	Question number										Total	Average
	A	B	C	D	E	F	G	H	I	J		
1	4	4	3	3	3	3	2	2	3	2	29	32.43
2	3	4	4	4	4	3	3	3	2	3	33	
3	5	4	3	5	3	2	2	3	2	2	31	
4	2	3	2	3	3	2	2	1	1	1	20	
5	4	4	5	5	3	2	2	3	2	3	33	
392	4	5	4	4	5	3	2	3	2	2	34	
393	3	5	4	3	4	3	2	2	3	3	32	
394	4	3	5	3	5	3	2	3	3	2	33	
395	2	3	3	3	3	2	2	3	3	3	27	
396	4	3	4	3	4	3	2	2	3	2	30	

### Evaluation of student learning effect

**Fig. 12:**
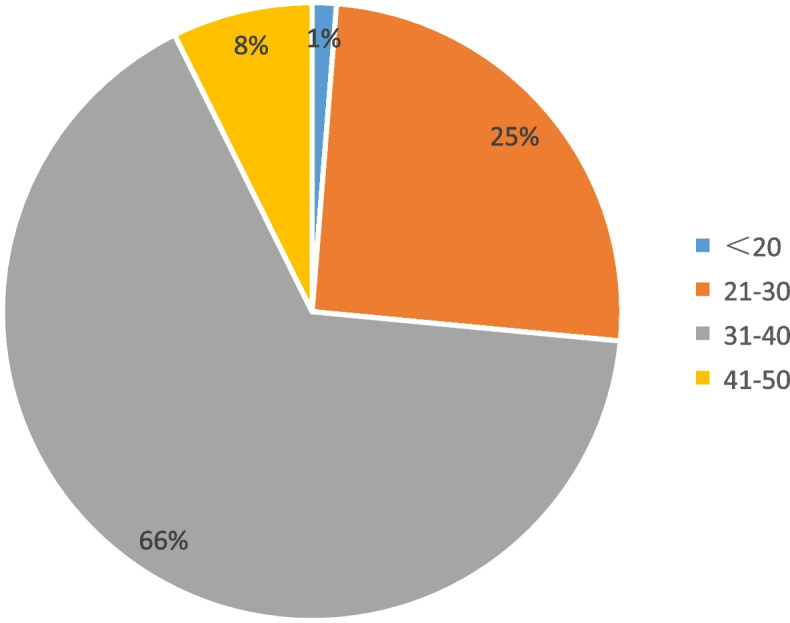
Learning effect score statistics

Figure 12 shows the learning effect scores of the 396 students in this survey. As shown in Fig. 11, the mean score of this survey was 32.43. Moreover, only 1% of students scored between 20 and below, 25% between 21 and 30, 66% between 31 and 40, and 8% between 41 and 50. To sum up, most students have learning effect scores ranging between 31 and 40.

### Development of dance teaching method based on knowledge concealment

Different teaching schemes are adopted according to the student’s scores and the method of knowledge hiding. In addition to offline teaching, different learning plans are adopted for students with different scores. Specifically, students who score was observed between 41 and 50 have relatively strong learning and understanding abilities, therefore, only the offline teaching method can be used. For students, with observed scores between 31 and 40, their dance foundation is relatively good. Still, their advanced dance ability is relatively weak, so the offline and online dance advanced video teaching method can be adopted. This should be noted that all students with 21–30 scores and 20 points or less have little advanced dance ability. The dance foundation is relatively weak so basic action teaching can be adopted offline and online dance. The specific teaching methods, in terms of learning method, and learning effect, and the criteria are shown in Table 7.

**Table 7:** Dance teaching methods based on knowledge concealment

Score	Learning effect description	Learning method	Learning effect
< 20, 21–30	Poor foundation, no advanced ability	Offline + dance basic video online teaching	Improve the foundation of dance
31–40	The foundation is good, and the advanced ability is poor	Offline + dance advanced video online teaching	Improve the ability to advance the dance
41–50	Good foundation, strong advanced ability	Offline teaching	Normal study

## The design of an online teaching system based on edge computing

### System architecture design

**Fig. 13:**
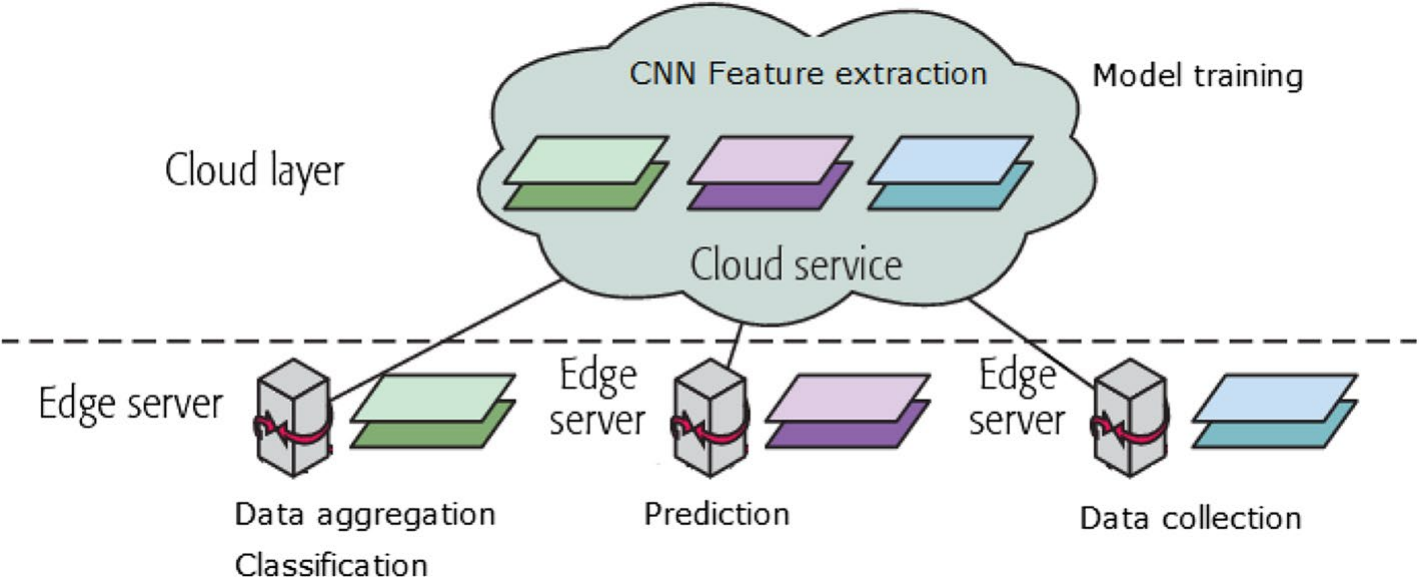
Architecture diagram of the online teaching system

The architecture of the online teaching system based on edge and cloud computing platform is shown in Fig. 13. The resource layer is implemented over the cloud platform due to the fact that it will needs significant amount of storage and also computational power when training a machine learning model. The interaction layer is implemented as a separate module over the edge server so that basic computation over the collected data can be done locally and only the essential data is transferred over the network to the cloud. In general, the capability of the cloud and edge server architecture as well as the individual application needs will determine how the CNN model is divided into sub-problems that may be executed on edge-cloud platform. The layers of the CNN model that demand a lot of processing power or have strict latency requirements might be divided, for example. Therefore, we divide the CNN model so that the earliest convolutional layers, which extract features, that need a lot of processing power, is executed on the cloud. Further layers that carry out categorization or prediction, as they require less computing power, operate on an edge server.

Edge servers are installed in IoT gateways at the edge layer to handle data that has been gathered. In the cloud server, we initially trained the deep learning networks. We split the learning networks into two after the training phase. The lower layers close to the input data are included in one section, while the higher levels close to the output data are included in another section. For processing offloading, we deploy the component with lower levels onto edge servers and the component with higher layers into the cloud. As a result, the edge servers’ first layer receives the gathered data. As the input data for the upper levels, the edge servers load the intermediate data from the lower layers and then transport it to the cloud server. The system architecture’s core is the edge computing layers. Among them, the edge layer mainly includes intelligent cameras and intelligent terminals, which can enhance the situational perception ability of the platform. It is mainly used to provide data support for collecting and uploading the dance movements of teachers and students, key frame extraction, and dance movement correction. The Edge computing layer is a network edge node set near the object or the data source, which can provide the image cache and rendering calculation for the teaching portal and gather and process the data collected by the sensing layer.

**Fig. 14:**
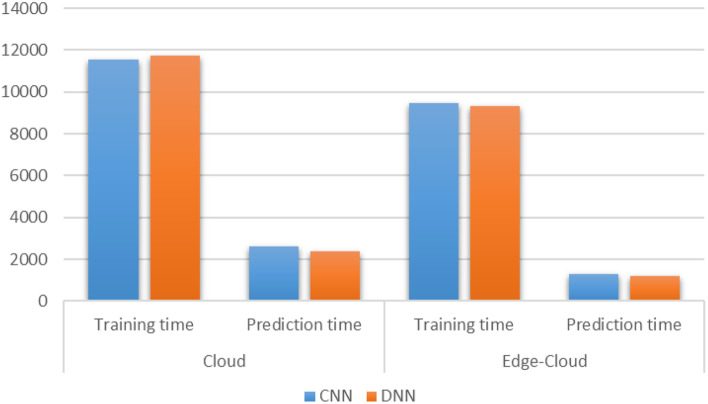
Results of CNN and DNN models for cloud only and edge-cloud platform in terms of training and prediction times [time is mentioned in seconds and the smaller values are better than the larger ones]

**Fig. 15:**
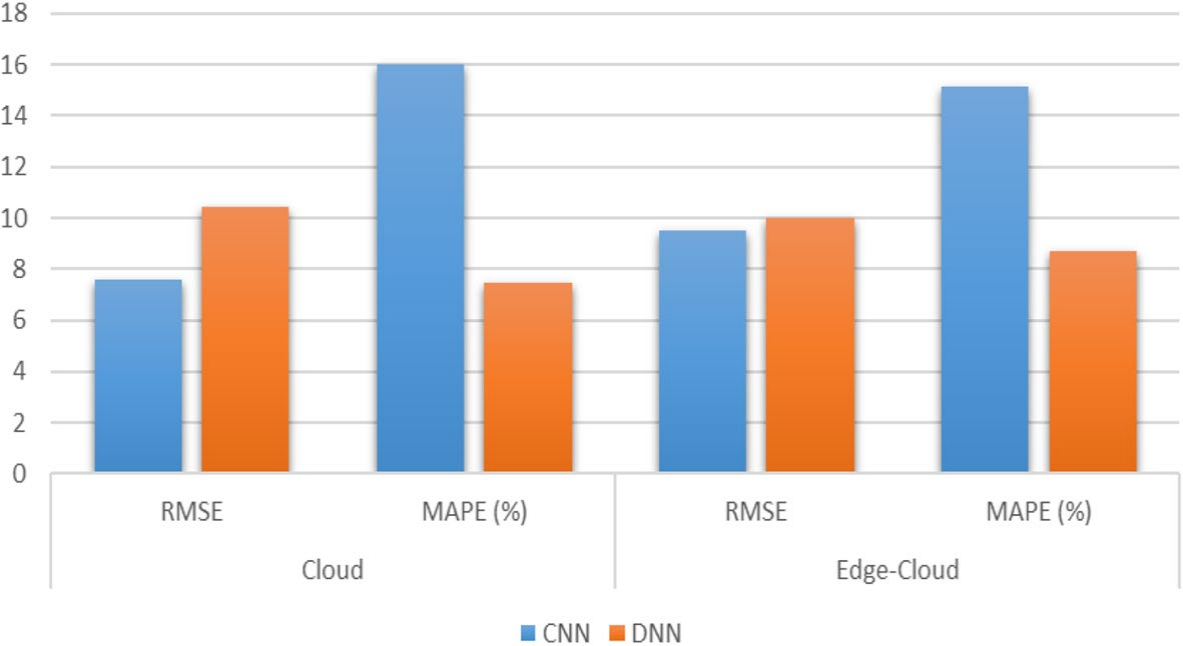
Results of CNN and DNN models for cloud only and edge-cloud platform in terms of RMSE and MAPE [the smaller values are better than the larger ones]

Setting the edge computing layers helps improve the platform’s overall processing performance and intelligence level. It gives full play to the advantages of edge computing mode in alleviating the pressure of network bandwidth and enhancing the service response-ability. Putting the data filtering function on the edge node server or even pushing it directly to the intelligent terminal can significantly reduce the amount of data transmitted by the network. Computing functions such as face recognition, attention model, and dance motion detection are deployed at the edge nodes to support the platform’s intelligent operation and refined training and management. Providing edge caching and video rendering calculation for HD video transmission can significantly reduce the latency and help improve the teaching experience. By enabling the platform scene perception, on-site teaching and online teaching can be effectively combined to improve the comfort and safety of the training site. The results are shown in Figs. 14 and 15. Figure 14 shows the results of CNN and DNN models for cloud only and edge-cloud platform in terms of training and prediction times. The time is mentioned in seconds and the smaller values are better than the larger ones. Similarly, Fig. 15 shows the results of CNN and DNN models for cloud only and edge-cloud platform in terms of RMSE and MAPE. Note that the smaller values for RMSE are better than the larger ones and vice versa.

### Application effect

**Fig. 16:**
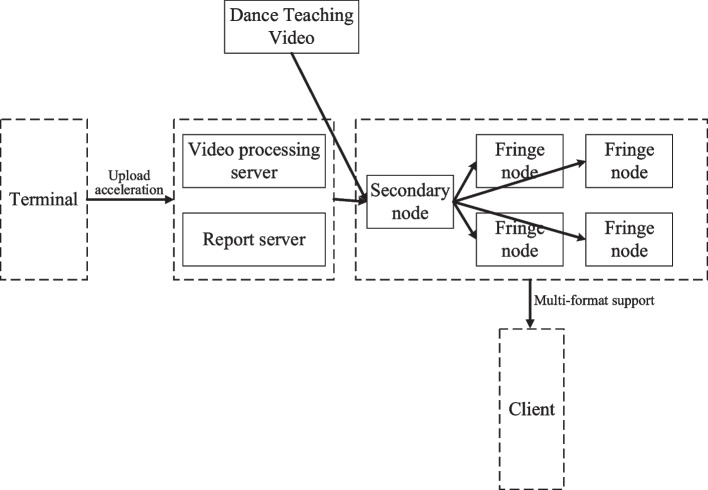
The CDN rubbings of the online teaching platform

The application of edge computing can effectively improve students’ online learning experience, mainly as follows:

1. Using base station caching, transparent cache caching, and other technologies in edge computing mode, training content can be cached to the network’s edge so that students can obtain learning content nearby and avoid repeated transmission of teaching content.
2. The mobile content distribution network in edge computing mode selects appropriate code rate and congestion control strategies for video transmission. It can realize the efficient transmission of ultra HD video and enable mobile devices to obtain the same traffic-carrying capacity as traditional Internet devices. And enable tens of thousands of students to simultaneously use different devices to participate in the live course broadcast, as shown in Fig. 16.
3. The VR/AR image rendering processing transferred to the network edge nodes can enhance the rendering effect, reduce the storage requirements of the terminal devices, but also improve the service response speed.

## Conclusions

This paper proposes an online dance learning method based on "video key frame extraction + human key point position extraction + action correction," which can identify and correct dance movements during estimation. We implemented the proposed scheme over an edge-cloud architecture that help in reducing the training duration and improves the response time. The main conclusions are as follows: (i) The accuracy and extraction speed of the dance teaching video key frame on the deep neural network is better than the traditional algorithms. The accuracy and recall rate is above 92%. The accuracy of human key point position prediction in the fully convolutional network based on grid coding is more than 90%. The instruction vector can correct the students’ dance movements correctly. (ii) The dance action correction method based on artificial intelligence can realize the online correction of students’ dance movements. (iii) Through the questionnaire survey, we can understand the effect of students’ dance learning and develop a reasonable dance teaching program based on knowledge concealment. (iv) The edge and cloud computing mode can effectively reduce the transmission delay of network dance teaching content, provide students with richer and faster teaching content, and significantly improve the online learning experience.

## Data Availability

The datasets used and/or analyzed during the current study are available from the corresponding author on reasonable request.
